# Colouring cryo-cooled crystals: online microspectrophotometry

**DOI:** 10.1107/S0909049509001629

**Published:** 2009-02-25

**Authors:** John McGeehan, Raimond B. G. Ravelli, James W. Murray, Robin Leslie Owen, Florent Cipriani, Sean McSweeney, Martin Weik, Elspeth F. Garman

**Affiliations:** a EMBL, 6 rue Jules Horowitz, 38042 Grenoble, France; b Biophysics Laboratories, School of Biological Sciences, Institute of Biomedical and Biomolecular Sciences, University of Portsmouth, Portsmouth PO1 2DY, UK; c Section Electron Microscopy, Department of Molecular Cell Biology, Leiden University Medical Center (LUMC), PO Box 9600, 2300RC Leiden, The Netherlands; d Laboratory of Molecular Biophysics, Department of Biochemistry, University of Oxford, South Parks Road, Oxford OX1 3QU, UK; e Imperial College, Exhibition Road, London SW7 2AZ, UK; f ESRF, 6 rue Jules Horowitz, 38043 Grenoble, France; g Laboratoire de Biophysique Moléculaire, Institut de Biologie Structurale, Jean Pierre EBEL, 41 rue Jules Horowitz, 38027 Grenoble Cedex 1, France

**Keywords:** radiation damage, macromolecular crystallography, online microspectrophotometry

## Abstract

X-rays can produce a high concentration of radicals within cryo-cooled macromolecular crystals. Some radicals have large extinction coefficients in the visible (VIS) range of the electromagnetic spectrum, and can be observed optically and spectrally. An online microspectrophotometer with high temporal resolution has been constructed that is capable of measuring UV/VIS absorption spectra (200–1100 nm) during X-ray data collection. The typical X-ray-induced blue colour that is characteristic of a wide range of cryo-conditions has been identified as trapped solvated electrons. Disulphide-containing proteins are shown to form disulphide radicals at millimolar concentrations, with absorption maxima around 400 nm. The solvated electrons and the disulphide radicals seem to have a lifetime in the range of seconds up to minutes at 100 K. The temperature dependence of the kinetics of X-ray-induced radical formation is different for the solvated electrons compared with the disulphide radicals. The online microspectrophotometer provides a technique complementary to X-ray diffraction for analysing and characterizing intermediates and redox states of proteins and enzymes.

## Abstract 2

A portable and readily aligned online microspectrophotometer that can be easily installed on macromolecular crystallography beamlines is described. It allows measurement of the spectral characteristics of macromolecular crystals prior, during, and after the X-ray diffraction experiment.

## 1. Introduction

‘So do we understand how enzymes work?’ is the title of a mini-review written by David Blow (2000 ▶). Starting with a reference to the crystallization of haemoglobin 160 years ago, it ends with describing our decade, where structural biology has become a mature branch of science, resulting in amazingly rapid progress of qualitative analysis of mechanism based on accurate structural data. ‘Superb papers have been written for the purpose of adding a single (curly) arrow to a cartoon’: the time of quantitative analysis and predictions of reaction rates is, however, still to come (Blow, 2000 ▶). He expected that, as computational power advanced, ‘we shall come back soon to tackling the detailed and fundamental questions of enzyme mechanism’.

The development of methodology and equipment to obtain data complementary to the static macromolecular X-ray structure could, in addition to progress in computational speed and resources, help the structural biologist to obtain a more quantitative understanding of the function of a macromolecule. In fact, the last few years have brought significant progress in analyzing crystals with techniques such as ultraviolet–visible light (UV/VIS) absorption spectroscopy, fluorescence spectroscopy (Weik *et al.*, 2004 ▶), Raman spectroscopy (Carey, 2006 ▶), electron magnetic resonance (Hogbom *et al.*, 2003 ▶), extended X-ray absorption fine structure (Pushkar *et al.*, 2008 ▶), X-ray absorption near-edge structure, or a combination thereof (*e.g.* Hough *et al.*, 2008 ▶). It is believed that the synergy between structural biology and these complementary techniques will lead to a deeper and more quantitative understanding of protein function (De la Mora-Rey & Wilmot, 2007 ▶).

The use of UV/VIS spectroscopy to complement and enhance X-ray crystallography is not new, and was initially developed for time-resolved studies using the Laue (Duke *et al.*, 1991 ▶) and Weissenberg (Gouet *et al.*, 1996 ▶) methods. Its common function is to confirm the identity of reaction intermediates within a crystal: different devices have been used successfully offline for some time (Hadfield & Hajdu, 1993 ▶; Bourgeois *et al.*, 2002 ▶; Ursby *et al.*, 2000 ▶). Only more recently have online microspectrophotometers become available at modern synchrotron macromolecular crystallography (MX) beamlines (Sakai *et al.*, 2002 ▶). Excellent reviews have been written describing the use of both off- and online devices (Bourgeois & Royant, 2005 ▶; De la Mora-Rey & Wilmot, 2007 ▶).

In this paper we will describe in detail the online device that has been developed for and implemented at the MX beamlines of the European Synchrotron Radiation Facility (ESRF, Grenoble, France). We initiated its development after cumbersome experiments (Murray & Garman, 2002 ▶; Weik *et al.*, 2002 ▶) where crystals were transferred back and forth between the beamline and an offline microspectrophotometer (Bourgeois *et al.*, 2002 ▶). Mirror lenses were custom designed to allow the incorporation of a spectrophotometer into the crowded space around the sample holder. The initial online set-up was used from 2003 to 2005 [for example, in research reported by Dubnovitsky *et al.* (2005 ▶)] and had a large working distance (lens to sample = 50 mm), allowing easy access to the goniometer for manual mounting and dismounting of the crystal. With the wide-scale automation of the ESRF MX beamlines (Arzt *et al.*, 2005 ▶; Beteva *et al.*, 2006 ▶; Cipriani *et al.*, 2006 ▶), a second-generation sample-changer-compatible online microspectrophotometer was designed [used, for example, by Macedo *et al.* (2009 ▶)], based on smaller lenses and a smaller working distance (lens to sample = 35 mm). Details of the design as well as plans for future generations of online microspectrophotometers are given in §2. The availability of both devices has been advertised to the MX community and they have been made accessible to external users, who have provided valuable input for the improvement of the set-up.

Whereas the external users of our devices have focused on verifying that the crystal under study still contained the desired intermediates during the acquisition of crystallographic X-ray data, we also started to explore other areas of crystallography. These include studies of macromolecular radiation chemistry (Southworth-Davies & Garman, 2007 ▶), automation of crystal alignment (Vernede *et al.*, 2006 ▶), development of ‘kinetic crystallography’ protocols (Colletier *et al.*, 2007 ▶) and the use of UV-damage for phasing (Nanao & Ravelli, 2006 ▶). However, a detailed description of the device has so far not been published.

In the following section of this paper, a full description is given of the online microspectrophotometer that has been used over the last few years at the ESRF, as well as some ideas and prototypes for future generations of our device. Observations such as the X-ray-induced blue colour seen in many samples for which we present evidence that this is due to trapped electrons, and the measurement of the lifetimes of X-ray-induced species are described in §3, in addition to some observations of X-ray-excited optical luminescence (Rogalev & Goulon, 2002 ▶). Finally, the possible origin of X-ray-induced colourization of vitrified macromolecular crystalline samples is discussed.

## 2. Instrumentation

**Figure 1:**
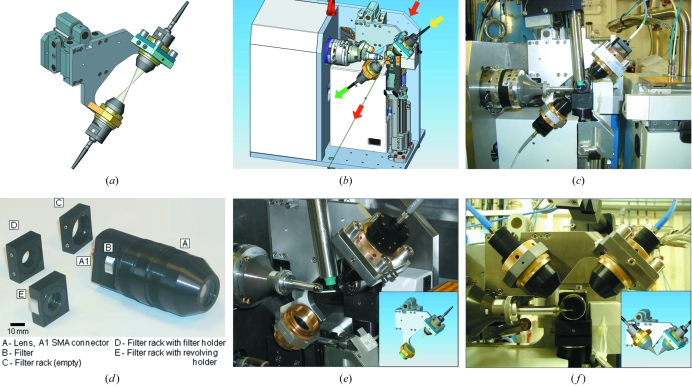
Design of the online microspectrophotometer. A new compact and portable microspectrophotometer was designed specifically as a module for the standard ESRF MX diffractometers. Two identical catadioptric objectives were mounted on a solid bracket that was in turn bolted to a high-precision *XYZ* translation stage (*a*). The mounting for the upper lens (light blue) has recessed micro-adjustment screws for aligning the two objectives to each other (focal cones depicted in light grey). Four locking nuts, one on each corner of the locking plate (dark blue), maintain this alignment. The rear of the translation stage (not visible) has a drilled recess to engage an alignment bar that is fixed permanently to the back plate of the diffractometer. This ensures correct and rigid docking, and the installation is completed by tightening two simple bolts. A CAD drawing of the instrument mounted on a MD2M diffractometer is shown in (*b*). The path of the X-ray beam is highlighted as red arrows and can be seen to intersect with the light path of the microspectrophotometer (yellow and green arrows). A typical installation is shown on ID14-2 (*c*). The objectives are mounted at 45° with respect to the ϕ axis to allow access for the cryostream, the X-ray fluorescence detector and the sample changer. A photograph of the device objective lens and accessories is shown in (*d*). A lens is shown complete with a rotating filter holder in place. The first mirror is visible on the front of the lens while the second larger mirror is situated internally at the rear of the lens. Fine threads have been machined onto the objective casing and allow positional adjustment and locking onto the mounting support (*a*). Filter holders can be inserted and removed while the instrument is installed on the beamline and offer the opportunity to adapt both the input and output transmission to match the experiment. The rotating filter holder offers the opportunity to insert polarizers into the light path. (*e*) Inset: a CAD drawing of a single objective design is shown. This set-up is essentially equivalent to the standard design except the second lower-objective has been replaced by a concave mirror. Both input and output transmission was achieved using a bifurcated fibre connected to the top lens. (*e*) Photograph: a prototype of this arrangement was constructed and successfully installed on ID14-2 ( *f* ). Inset: a dedicated fluorescence set-up was designed in order to optimize the collection of emission spectra while reducing contamination from the excitation source. ( *f* ) Photograph: a prototype was constructed and installed on ID14-4.

The design of the current online microspectrophotometer is shown in Fig. 1(*a*) and its integration onto the beamline in Figs. 1(*b*) and 1(*c*) ▶. Fig. 1(*b*) shows the intersection of the UV/VIS light path with the X-ray beam. The light source can be chosen to match the spectral region of interest. In most of our studies, a combined deuterium-halogen lamp was used (DH2000, Ocean Optics, http://www.oceanoptics.com/), coupled through an SMA-terminated fibre (between 50 and 600 µm in diameter and 2 m long, supplied by Ocean Optics) to a custom-made mirror lens system developed at the EMBL and commercialized by ACCEL/MAATEL. The light path within the illumination and detection objectives is similar to that in the Ealing reflective objective as shown in Fig. 1 of Hadfield & Hajdu (1993 ▶): light is refracted onto the first mirror by the entrance optical element and reflected onto the second internal convex mirror before exiting *via* an optical fibre connection. The second mirror blocks out 20% of the light entering the lens.

A second identical objective is positioned symmetrically around the sample position to pick up the transmitted light (Fig. 1 ▶). The symmetric set-up causes the blind spots of each objective to overlap. The mirror lens reflects over the full UV–visible–near-IR wavelength range and focuses the entire spectrum from a fourfold magnified collection field to the output fibre optic without chromatic aberration.

Originally, large-diameter (80 mm) lenses were used in order to allow a long working distance of 50 mm [see Fig. 1 of Nanao & Ravelli (2006 ▶)], and these could be installed on beamline ID14-4. Although these lenses provided optimal access to the goniometer for manual mounting of the vitrified sample, the size and weight of these lenses required a heavy support that by itself restricted the amount of free space around the goniometer. This arrangement was abandoned after the automation of the beamlines, as the Grenoble sample changer required free access to the goniometer. Instead, smaller and lighter lenses were designed, with a working distance of 35 mm of each lens to the sample position. The outer diameter of these lenses is only 40 mm, whereas the front of the lens is 17 mm (Fig. 1*d* ▶). The internal parts of the lens are protected from dust by a UV grade silica entrance window. A filter port is available to introduce half-inch diameter (5 mm thickness) glass coloured filters, interference filters or polarizers. The polarization angle can be tuned by the use of a filter rack with revolving holder (Fig. 1*d* ▶). This second microspectrophotometer can be easily installed for temporary use on any of the ESRF MX beamlines equipped with the standard minidiffractometer (MD2M) set-ups, and experiments have been performed on beamlines ID14-2, ID14-4 and ID23-1.

The mechanical support allows pre-alignment of both lenses with respect to each other. The distance of each lens to the focal point can be tuned separately using a fine-pitch thread on the lens and a lock ring. The radial adjustment is performed with only one lens, using a sliding plate with sprung *XY* fine-pitch stop screws and locking nuts. The support with the mutually aligned lenses can be mounted directly onto a minidiffractometer. The support is compatible with X-ray fluorescence detection, a mini-κ goniometer (used at κ = 0°), and a CCD or pixel-area detector used at minimal crystal–detector distance as well as with nitrogen and helium cryostreams. The focal point of both lenses is aligned towards the sample position using a high-precision high-stability manual *XYZ* translation stage. This stage has an alignment range of 2 mm in all directions and a resolution of <1 µm. The alignment procedure utilizes the minidiffractometer on-axis camera to view a spot from the halogen source projected from the uppermost lens onto a metal foil mounted at the sample position. Fine adjustment is made by maximizing the intensity of halogen light transmitted through a 50 µm pinhole placed at the sample position while measuring real-time transmission compared with a reference spectrum. A trained scientist can align the instrument in about 20 min, although the initial mounting of the device on the beamline by the local contact can take an additional hour. With the microspectro­photometer *in situ*, a cone with a maximum opening angle of 125° can be used before shadowing is observed on the detector. For an ADSC Q315r detector, this corresponds to a minimum sample to X-ray CCD detector distance of 82 mm. The microspectrophotometer stand does not reduce the minimum crystal-to-detector distance of 35 mm for the MD2M.

The receiving objective is coupled through a wider optical fibre (typically 600 µm diameter) to a high-resolution spectrophotometer (HR2000, Ocean Optics). The spectrophotometer consists of a 50 µm entrance slit, a 600 lines mm^−1^ grating and a linear 2048-element CCD array. It covers the wavelength range 200–1100 nm with 0.5 nm resolution, has a dynamic range ratio of 2000:1, has a readout time of 10 ms, and is linked through a USB connection to a notebook computer that can be remotely controlled. The lamp shutter and the spectrophotometer are directly driven by the *OOIBase32* or *Spectrasuite* software suites (Ocean Optics). Following the collection of a dark current (halogen/deuterium lamp shutter closed) and a reference spectrum (light without the crystal in the light path), an appropriate integration time is selected based on the absorbance of the sample. Collection of continuous full spectra is possible at rates of up to 40 Hz while, in addition, several wavelength ranges can be monitored as a function of time. In addition to design and technical details, a full operating manual is available online.^1^

A single-lens set-up with a mirror could be used for UV/VIS measurements if the space around the sample is restricted. Fig. 1(*e*) ▶ shows the design and illustrates a prototype of such an arrangement. In this case both the probing and the transmitted light pass through the same objective. A bifurcated fibre guides the monitoring light into the lens, whereas the transmitted light is led through the other fibre arm towards the spectrophotometer. A concave mirror with a focal point at a position equivalent to that of the second lens of the more standard set-up and placed opposite the objective reflects the light back through the sample and into the objective. The crystal will subsequently absorb the light twice, resulting in reduced transmission. However, for studying X-ray-induced colour changes in transparent crystals, the set-up shown in Fig. 1(*e*) ▶ is adequate. The reflecting mirror for this prototype is somewhat large; however, we are optimistic that a smaller version of this mirror would work equally well, thus extending the compatibility to the MD2 microdiffractometer.

A bifurcated fibre in combination with a single lens from the standard set-up (as in Fig. 1*a* ▶) allowed the monitoring of optical luminescence from the crystal with a 0° face-on geometry, as used in the FLUMIX device developed by Klink *et al.* (2006 ▶). However, as the luminescence signal is, in general, rather weak, we designed an optimized 90° dual lens excitation-emission set-up (Fig. 1*f* ▶ ) as would be employed in a conventional fluorimeter. A smaller support was required compared with the transmission dual- or single-lens set-up described above. One lens is used to guide the light of an excitation source, in this case a laser, onto the crystal. In order to prevent saturation of the spectrophotometer by scattered light from the sample, a selective absorbance filter can be placed in the filter holder of either lens. Laser cut-off or notch filters are used in the second lens filter holder only.

In general, thinner crystals (maximum of approximately 100 µm in light beam path) had to be used as otherwise the absorption was too high. At the start of measurements on each sample, the solution or crystal was rotated and translated to find the optimum position where the background was low enough for a reasonable signal-to-noise level to be obtained. The presence of ice increased the background to the point where meaningful measurements could not be made, so cryo-cooling protocols that ensured no ice formation were essential.

Note that the Sakai *et al.* (2002 ▶) microspectrophotometer system at SPring-8 uses a halogen-tungsten light source with a double monochromator and a photomultiplier detector. This means that much thicker samples can be tolerated, up to an absorbance (optical density) of ∼5 compared with a maximum absorbance of ∼2 (optimally 0.5–1) for the system described here. However, owing to the lens system used, the wavelength range is limited to 350–800 nm and a complete wavelength scan takes 2 min compared with the 25 ms time resolution of our microspectrophotometer.

## 3. Materials and methods

All chemicals and proteins were obtained from Sigma (except where noted otherwise) at the highest available quality. The crystallization and cryo-protection conditions for chicken egg-white lysozyme (HEWL) are given by Murray & Garman (2002 ▶).

Apoferritin was used with no further purification and crystallized by hanging-drop vapour diffusion from a 1:1 mixture of 50 mg ml^−1^ ferritin and 0.1 *M* sodium chloride, 0.8 *M* ammonium sulfate, and 10 m*M* cadmium sulfate with 25% glycerol (*v*/*v*). The crystals did not require further cryo-protection and were flash-cooled to 100 K in the beamline cryostream.

*Bos taurus* (bovine) trypsin (23 kDa) was used without further purification. Orthorhombic (*P*2_1_2_1_2_1_) crystals were grown at room temperature in 23–25% (*w*/*v*) polyethylene glycol (PEG) 8000, 0.2 *M* ammonium sulfate, 0.1 *M* Tris buffer pH 8 and 100 m*M* benzamidine (inhibitor). An initial protein concentration of 15 mg ml^−1^ was employed.

N9 neuraminidase from avian influenza (Noddy Tern) was grown, purified and crystallized as described previously (Laver *et al.*, 1984 ▶). Crystals were grown by hanging-drop vapour diffusion from a mother liquor of a 1:2 mixture of 3.0 *M* K_2_HPO_4_ and 1.4 *M* KH_2_PO_4_. These crystals were serially cryo-protected *in situ* (Garman, 1999 ▶) up to a concentration of 40% glycerol (*v*/*v*) in 10% steps, in the mother liquor solution with glycerol replacing water, over a total of 3 min and then flash-cooled in a 100 K nitrogen stream (600 series, Oxford Cryosystems, Oxford, UK).

**Figure 2:**
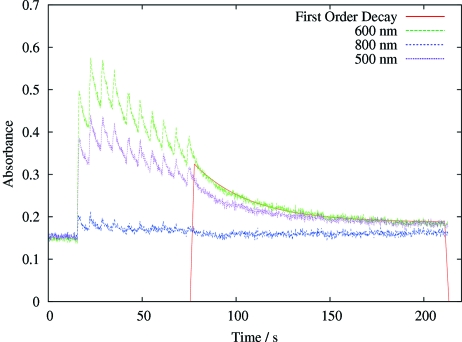
The absorption of 40% glycerol solution irradiated for beam pulses of 1 s followed over time at 500 nm, 600 nm and 800 nm. The decay of the signal at 600 nm after the final pulse is fitted to a first-order exponential, also shown. The dose per 1 s of beam irradiation was estimated to be 4 × 10^4^ Gy, giving a total of 4 × 10^5^ Gy for the 10 s of data shown here.

Solutions of glycerol and water ranging from 100% to 40%(*v*/*v*) glycerol were produced by serial dilution of 100% glycerol. These were mounted in 300–500 µm diameter rayon cryo-loops and cryo-cooled to 100 K in the beamline cryostream as for the crystals. Full spectra were recorded continuously as irradiation proceeded. The solution spectra were recorded using the halogen lamp with an integration time of 10 ms and threefold averaging. The dose per 1 s of beam irradiation was estimated to be 4 × 10^4^ Gy, giving a total of 4 × 10^5^ Gy for the 10 s of data shown in Fig. 2 ▶. A 25 µm light spot was used to monitor the spectral changes induced in the samples by a 200 × 200 µm X-ray beam. Solutions of PEG 400, MPD and glucose over a range of concentrations were tested in an analogous manner. For glycerol, 30% glycerol with 1.5 *M* HCl and with 1.5 *M* NaOH were also tested.

Spectra collected at ID14-4 on the apoferritin, N9 and lysozyme crystals were obtained using the combined halogen-deuterium light source with 25 µm probe spot size, 100 ms integration time and threefold averaging. The 13.2 keV X-ray beam was 200 × 200 µm and 20 times attenuated. The dose per 1 s of beam irradiation was estimated to be 4 × 10^4^ Gy using the program *RADDOSE* (Murray *et al.*, 2004 ▶), giving a total of 1.2 × 10^6^ Gy for the 30 s of exposure to which these crystals were subjected.

For the temperature-dependent absorption spectroscopy studies, a crystal of bovine trypsin (dimensions 300 × 50 × 50 µm) was soaked for 20 s in a cryo-protectant solution containing 24% (*w*/*v*) PEG8000, 0.2 *M* ammonium sulfate, 0.1 *M* Tris buffer pH 8 and 15% glycerol before cryo-cooling in the cryostream operating at 100 K on ID14-4. The long-axis dimension of the crystal was aligned parallel to the spindle axis. Ten consecutive exposures to the unattenuated X-ray beam [energy: 13.2 keV; slits: 50 µm (horizontal) and 100 µm (vertical)] were applied, each lasting for 1 s and separated in time by about 4 s. Full-wavelength-range absorption spectra were recorded continuously at a rate of 2 Hz. The crystal was then translated horizontally along the spindle axis by 70 µm and the temperature of the cooling device raised to 130 K at 360 K h^−1^. This previously unexposed part of the crystal was not damaged by diffusing radicals as assessed by the absence of characteristic absorption signals of radicals. The irradiation procedure was repeated at 130 K with simultaneous spectroscopic data collection. The crystal was then translated again horizontally by 70 µm, the temperature raised to 160 K at 360 K h^−1^ and the irradiation procedure with spectroscopic measurements repeated at 160 K. Again, for this previously unexposed part of the crystal, no signs of damage owing to putative radical diffusion from the part exposed at 130 K were observed. The absorbed dose per 1 s X-ray pulse was 4.4 × 10^4^ Gy as calculated with the program *RADDOSE*.

X-ray-induced optical luminescence measurements were obtained on beamline ID14-2 as described above except utilizing a single lens. A 600 µm-diameter optic fibre, producing a 150 µm light collection field, was used to connect directly to the HR2000 spectrophotometer without filters. The X-ray beam was 200 × 200 µm and centred to be coincident with a cryo-cooled (100 K) bovine trypsin crystal and the focal spot of the collecting lens. Spectra were measured over the range 200–1100 nm using an extended integration time of 30 s during exposure to an unattenuated X-ray beam. A dark spectrum (no X-rays) was recorded to ensure that the background subtraction was stable.

All spectra were recorded using the *OOIBase32* software (Ocean Optics). Each collected spectrum consisted of 2048 absorbance points between the wavelengths 200 and 1100 nm. The spectra were processed with Perl, and *GNUPLOT* was used to fit the background as well as Gaussian functions to the broad slightly asymmetric absorbance peaks for the solution spectra. For each solution spectrum, a maximum as well as full width at half-maximum (FWHM), σ and goodness-of-fit values were obtained, and were plotted against time. For some series, data collection was interrupted while data were written to disk, resulting in a single time gap of up to 10 s. For some experiments, 10 or 20 sequential 1 s exposures were collected, with 4 s gaps corresponding to the CCD detector readout time. This protocol resulted in a ‘saw tooth’ pattern of intensity against time at a particular wavelength (see, for example, Fig. 2 ▶).

Lifetimes of species were obtained by processing the raw spectra with Perl scripts, which read in the thousands of individual spectra and sorted them into a single two-dimensional matrix. This convenient format could easily be processed in *GNUPLOT*, *MATLAB*, *ORIGIN* *etc.* to generate a time series of points at a particular chosen wavelength. A first-order decay curve could then be fitted in *GNUPLOT* or *ORIGIN* for the time period corresponding to after the beam had been turned off.

## 4. Results

### 4.1. Trapped electrons

**Figure 3:**
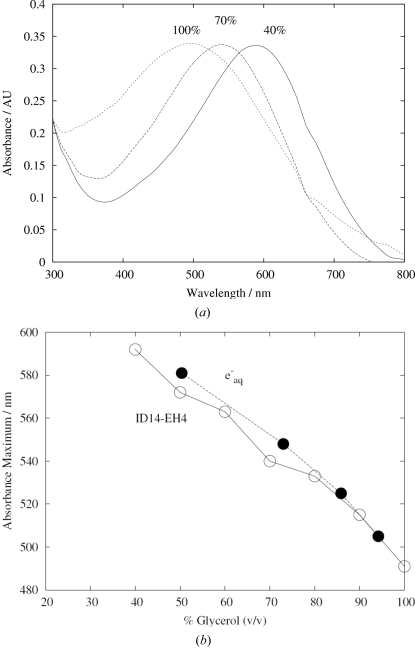
(*a*) Comparison of the (normalized) X-irradiated spectra of glycerol solutions of 40%, 70% and 100% concentration, showing the decrease in wavelength of the absorption peak with increasing glycerol concentration. (*b*) A comparison of our measurements of the absorption maximum of X-irradiated glycerol solutions at different concentrations, with values for the trapped electron in glycerol measured previously by Ershov & Pikaev (1968 ▶). The curve with filled circles refers to the literature values, and the curve with unfilled circles to our own measurements.

Measurements of the signature spectral peak of the trapped electron at around 600 nm were carried out for different concentrations of solutions of glycerol (Figs. 3*a* and 3*b* ▶), PEG 400 (peak observed between 583 and 615 nm but no systematic trend with concentration), MPD (no peak observed) and glucose (no peak observed). The 600 nm peak corresponds to the absorption of red light by the sample and accounts for the blue colouration observed in irradiated solutions and crystals. For glycerol, the results agree well with those presented by Ershov & Pikaev (1968 ▶): their measurements are also shown in Fig. 3(*b*) ▶. The wavelength of the absorption peak decreases monotonically with increasing glycerol concentration.

The analysis of the spectra of the solvated electron in glycerol gives a half life for the species of 21 s at 100 K (40% glycerol *v*/*v* at 600 nm: see Fig. 2 ▶). The saw-tooth pattern referred to above is evident, and it is interesting to note that the trapped electron peak saturates after only 3 s of irradiation, and that after the last irradiation the absorbance peak decays exponentially by a first-order process.

Trapped electrons were observed in alkaline (1.5 *M* NaOH, pH 14) and neutral glycerol solutions but not in acidic solutions. The only peak in the 1.5 *M* HCl/glycerol (pH 0.1) spectra was at 370 nm, which is likely to be from the Cl_2_ 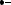 ion chloride radical (Atinault *et al.*, 2008 ▶). It may be that under these conditions the lifetime of the electron is shorter than the minimum measureable by our apparatus.

### 4.2. Disulfide-containing proteins

**Figure 4:**
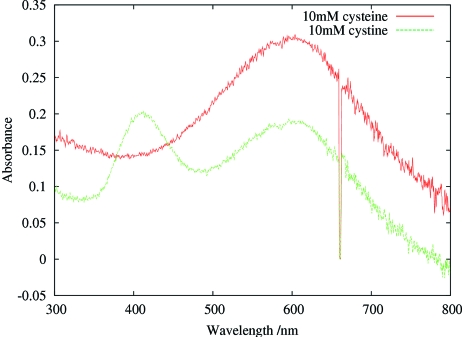
Graph showing the absorption spectra of loop-mounted X-irradiated solutions of cysteine (10 m*M*) and cystine (10 m*M*) in 30% glycerol with 1.5 *M* NaOH for 1 s of beam irradiation (dose approximately 4 × 10^4^ Gy). Both solutions show absorption maxima at around 550 nm from the trapped electrons, but only the cystine solution has an absorption peak at 400 nm corresponding to the disulfide radical anion.

Using the offline spectrophotometer installed at the ESRF cryobench (Bourgeois *et al.*, 2002 ▶), we observed that irradiated crystals of lysozyme and of acetylcholinesterase had an absorption peak at around 400 nm (Murray & Garman, 2002 ▶; Weik *et al.*, 2002 ▶). From previously reported work (Favaudon *et al.*, 1990 ▶), this was identified as the disulfide radical anion (RSSR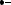) that is elongated by 0.7 Å with respect to a neutral disulfide bond (Weik *et al.*, 2002 ▶). This peak was present in irradiated crystalline samples of the disulfide-containing proteins that were examined: lysozyme (244 m*M* S—S bond concentration), bovine trypsin (192 m*M*), N9 neuraminidase (120 m*M*), but not in apoferritin which, although it has no disulphides, has two cysteines (52 m*M* cysteine) per monomer. The concentrations of the disulphide bonds in the crystals were calculated from known structures and crystal space-group parameters. The disulfide radical anion peak is visible in irradiated samples of cystine solutions (oxidized cysteine which is two cysteine residues linked by a disulphide), as expected, but not in the irradiated solutions of cysteine (see Fig. 4 ▶), which has no disulfide bond (Southworth-Davies & Garman, 2007 ▶).

**Figure 5:**
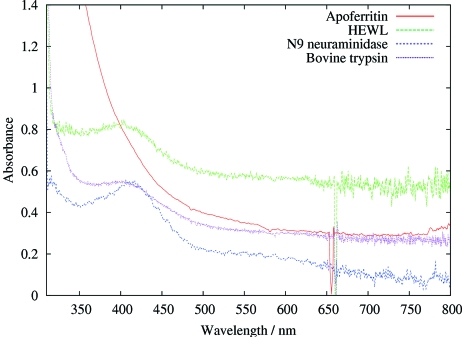
Spectra from X-irradiated crystals of apoferritin, N9 neuraminidase, HEWL and bovine trypsin. All but apoferritin show a peak around 400 nm corresponding to the disulfide radical anion. The broad absorption of the apoferritin from 500 nm into the UV region is probably due to the presence of residual iron in the protein crystal. The dose per 1 s of beam irradiation was estimated to be 4 × 10^4^ Gy, giving a total of 1.2 × 10^6^ Gy for the 30 s of exposure to which these crystals were subjected.

A superposition of the spectra from irradiated crystals of these proteins is shown in Fig. 5 ▶ (bovine trypsin, lysozyme, N9, ferritin); the absorption owing to the 400 nm disulphide radical anion is clearly visible. As for the glycerol series shown in Fig. 2 ▶, the absorption peaks reach a maximum within the time frame of the experiment, and then the final peak appears to decay by first-order kinetics.

Lifetimes were calculated from such spectra, but proved to be rather irreproducible. For instance, for the HEWL crystal, the half-life of the 400 nm peak as measured using the offline microspectrophotometer (Murray & Garman, 2002 ▶) was 79 min, whereas with the online device a value of 126 s was obtained. We currently have no satisfactory explanation for these disparate results, but it could be that there is a fast and slow decay component, and that we were sensitive to the former using the beamline spectrometer and the latter on the offline device where the time between irradiation and measurement was approximately 20 min rather than immediately following the closure of the shutter.

### 4.3. The effect of temperature

**Figure 6:**
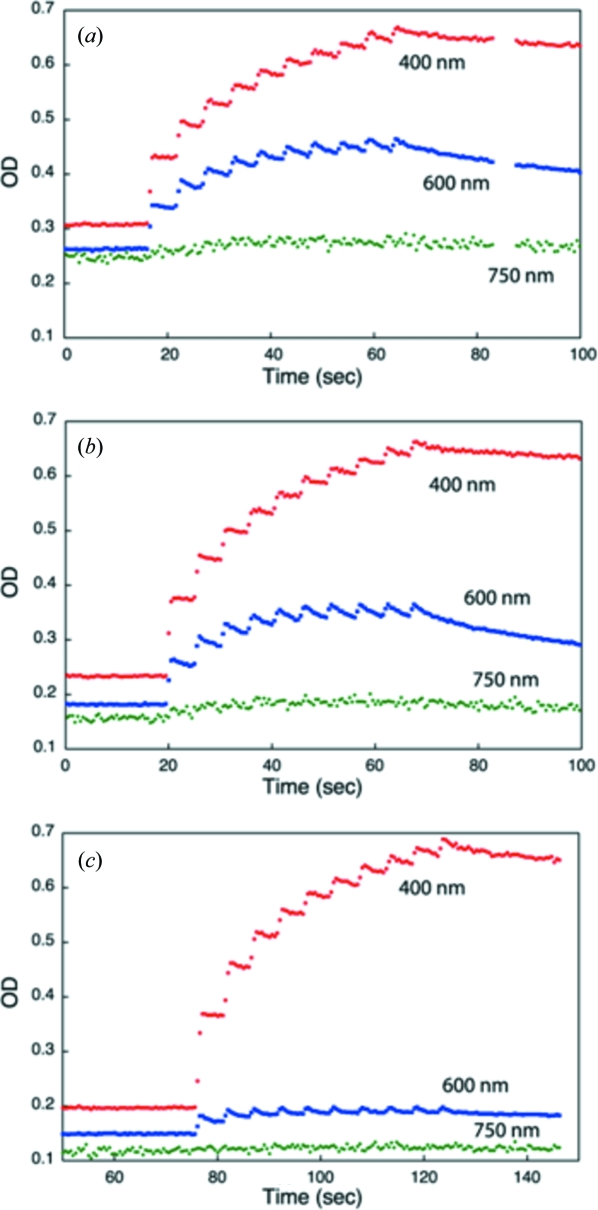
Temperature-dependence of disulfide radical lifetimes. The optical density (OD) of an orthorhombic trypsin crystal irradiated for ten beam pulses of 1 s followed over time at 400 (red), 600 (blue) and 750 nm (green) at (*a*) 100, (*b*) 130 and (*c*) 160 K. The decay of the signals at 400 nm after the final pulses was fitted to a first-order exponential to extract disulfide-radical lifetimes. The absorbance at 600 nm corresponds to the presence of trapped electrons; the one at 750 nm is shown as a reference wavelength. The dose per 1 s of beam irradiation was estimated using *RADDOSE* (Murray *et al.*, 2004 ▶) to be 4.4 × 10^4^ Gy, giving a total of 4.4 × 10^5^ Gy for the 10 s of data shown here.

The lifetime of disulfide radicals as a function of temperature was investigated with flash-cooled orthorhombic bovine trypsin crystals. Ten consecutive X-ray irradiations at 100 K produced disulfide radicals, of which the absorption at 400 nm was followed as a function of time (Fig. 6*a* ▶). Upon the opening of the shutter, the absorbance at 400 nm increased instantaneously. During the 1 s X-ray exposure, the absorbance increased further, owing to the dose-dependent accumulation of disulfide radicals. In the subsequent period of 4 s without irradiation, the absorbance does not remain constant but decays. In the following periods with and without X-ray pulses, the absorbance again increases and decreases, respectively. From the decay after the final (tenth) X-ray pulse, a half life of around 300 s was obtained. Experiments on a previously unexposed part of the same crystal at temperatures of 130 and 160 K showed approximately the same decay rate (Figs. 6*b* and 6*c* ▶).

In contrast, the 600 nm trapped electron peak does show a temperature dependence (Fig. 6 ▶). The maximum absorbance reached is higher at lower temperatures even though the decay rates between exposures are, within the uncertainty of the measurements, constant for all temperatures (lifetimes are 26, 29 and 35 s at 100, 130 and 160 K, respectively).

### 4.4. X-ray-excited optical luminescence (XEOL)

**Figure 7:**
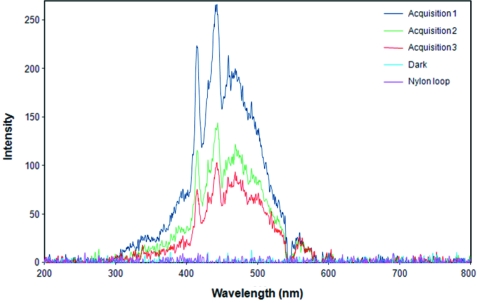
X-ray-induced luminescence from a cryo-cooled bovine trypsin crystal. Spectra were collected between 200–1100 nm; however, no signal was observed above 620 nm. Three consecutive 30 s acquisitions are shown from the same crystal. No fluorescence is detected from the nylon loop and the noise is comparable with the dark measurement.

Preliminary experiments demonstrated that an XEOL signal could be detected from cryo-cooled crystals during routine data collection. By increasing the microspectrophotometer CCD integration time and using longer X-ray exposures, sufficient counts were obtained to give good quality spectra. Some features were visible within the XEOL spectrum of a single trypsin crystal (Fig. 7 ▶). The full region 200–1100 nm was probed but the main observed luminescence signal was restricted to the visible region between 400 nm (violet) and 550 nm (pale green). Smaller contributions were detected towards the UV at 325 nm and towards the red at 575 nm. Areas of fine structure are evident despite the low signal-to-noise ratio, and major bands are clearly observed at 414 nm and 442 nm. Three consecutive 30 s measurements on the same crystal in the same orientation demonstrate a marked temporal reduction in luminescence as the exposures progress. For this 90 s continuous data collection the rate of reduction decreases during each consecutive 30 s measurement; however, this observed rate is not uniform between individual bands in the spectrum. The reduction of intensity is likely to be caused by radiation-induced damage to the optically active sites, an effect that has been observed in luminescent proteins (Berovic *et al.*, 2002 ▶). No luminescence was detected from the nylon loop, indicating that the XEOL signal originates either from the protein, the buffer or both (sample conditions as for the absorption measurements described above).

## 5. Discussion and perspectives

This paper describes a portable and readily aligned online microspectrophotometer that allows the spectral characterization of macromolecular crystals prior, during and after the X-ray diffraction experiment. The device, designed and commissioned in 2004, has been made publicly available to the general European macromolecular synchrotron community. It has proven to be a vital instrument for kinetic crystallographic studies as the X-ray beam can induce specific changes to particular sites that must be deconvoluted from the structural differences relevant to biological mechanism (Mees *et al.*, 2004 ▶; Colletier *et al.*, 2007 ▶, 2008 ▶; Burmeister, 2000 ▶; Weik *et al.*, 2000 ▶; Ravelli & McSweeney, 2000 ▶).

Ideally, a user would have a detailed knowledge of all the likely spectral changes of their system prior to X-ray data collection at a synchrotron. A monochromatic device such as that of Sakai *et al.* (2002 ▶) would then allow fast absorption changes at a particular wavelength to be followed up to high optical densities. However, in our experience, it is also greatly beneficial to have fast monitoring of the full spectrum, so that not only changes in the background signal but also novel transient and spectrally different radiation-damage-induced intermediates can be observed simultaneously.

There are a few constraints to consider if good absorption spectra are to be recorded online. An orientation of the crystal has to be found where blockage (*e.g.* by the loop material) or reflection (*e.g.* from the crystal surface) of the light are minimized. As the quality of the absorption spectra changes upon rotation of the spindle axis, spectra have to be collected at a reference angle if quantitative comparisons are to be made. With modern goniometers the same sample position can be achieved with high accuracy, allowing interleaved X-ray diffraction and spectroscopic data collection where spectra are taken solely at a defined crystal position and the spindle is returned to the reference angle between the collection of successive frames (Wilmot *et al.*, 2002 ▶). Alternatively, the appropriate data collection protocol could be determined from thin crystals held at a stationary orientation. For strong UV/VIS absorbers, such thin samples are essential. In terms of X-ray absorption, most crystals are highly transparent, making the damage induced within the crystal largely independent of crystal depth (Murray *et al.*, 2005 ▶). A thin sample could thus serve to establish an X-ray data collection protocol using the online microspectrophotometer, whereas a thicker crystal could subsequently be used to collect the best possible X-ray diffraction data.

Another important factor that determines the quality of the spectra obtained is how well the crystals have been vitrified. A good cryoprotectant must be chosen (Garman, 1999 ▶) and dry liquid and gaseous nitrogen should be used to prevent the deposition of ice particles on the sample. Badly vitrified and opaque crystals will show poor transmission of the monitoring UV/VIS light, in particular at short wavelengths. The measurement of either X-ray-induced optical luminescence or UV/VIS-light-induced fluorescence could overcome some of the problems caused by high sample optical density.

Two commonly observed X-ray-induced radicals have been characterized: the trapped electron and the disulphide radical. Their relative lifetimes at 100 K of 26 s and 300 s, respectively, are much longer than the typical exposure time on our brightest X-ray sources.

Trapped electrons have been identified in pure water (Hart & Boag, 1962 ▶), with an absorption maximum of 580 nm at room temperature. Taking the extinction coefficient as 18700 *M* ^−1^ cm^−1^ (Keene, 1964 ▶), a sample thickness of 100 µm and an OD of 0.3 (a typical value as can be seen from Fig. 2 ▶), the concentration of trapped electrons during irradiation is estimated to be around 2 m*M*. This absorption accounts for the bluish hue often observed marking the path of the X-ray beam in cryo-cooled crystals in loops. The absence of trapped electrons at acidic pH might be caused by the highly efficient scavenging by protons [hydrated electron + hydronium room-temperature rate constant is 2.3 × 10^10^ *M* ^−1^ s^−1^ (Buxton *et al.*, 1988 ▶)]. As described above, trapped electrons decay significantly faster than disulfide radical anions.

The absorption coefficient of the disulphide radical at between 410 nm and 425 nm, ∊_410–425_, is estimated to be 4000–9000 *M* ^−1^ cm^−1^ (Favaudon *et al.*, 1990 ▶). If the sample is assumed to be uniform, the thickness is taken to be between 50 and 100 µm and the absorbance of the irradiated crystal to be around 0.5, the concentration of this radical may be estimated at between 35 m*M* (using 4000 *M* ^−1^ cm^−1^ and 50 µm) and 8 m*M* (using 9000 *M* ^−1^ cm^−1^ and 100 µm). The concentration of disulfide bonds in an orthorhombic bovine trypsin crystal is 192 m*M*, which implies that as a proportion these concentrations of the radical would only be observable crystallographically if highly accurate high-resolution data were available. In general, X-ray-induced species can be detected spectroscopically long before they can be crystallographically observed.

We observed little temperature dependence of the S radical and solvated electron lifetime in the temperature range from 100 to 160 K (Fig. 6 ▶). However, the maximum ODs of the trapped electrons do show a clear temperature dependence (Fig. 6 ▶). The saw-tooth patterns of Figs. 2 ▶ and 6 ▶ can only partly be explained by considering a negative decay rate constant *k*_2_ in d[radical]/d*t* = *k*_2_[radical] and a positive build up rate *k*_1_ in d[radical]/d*t* = *k*_1_ when the beam is on. At least an additional third rate constant would be needed to describe the observation that the maximum OD was obtained after the second image was taken in the experiment shown in Fig. 2 ▶. Such a rate constant could be dose dependent, *e.g.* reflecting a reduction of the number of available sites for trapped electrons as these sites become damaged themselves. A full characterization and understanding of the reaction kinetics behind Figs. 2 ▶ and 6 ▶ awaits further experiments and analysis: the results plotted in Fig. 6 ▶ show that temperature would be an interesting parameter to include in these studies. For cryo-cooled (not transferred to oil) orthorhombic bovine trypsin crystals the amorphous solvent within the crystal channels becomes an ultraviscous liquid at its crystallization temperature of 185 K (Weik *et al.*, 2005 ▶). Figs. 6(*a*)–6(*c*) ▶ compare the radical concentrations and lifetimes at temperatures well below this value. Nevertheless, the temperature dependence of the kinetics of the disulfide radical, located within the protein region, and that of the trapped electrons, located in the solvent, are strikingly different.

Since the installation of high-magnification visualization cameras on MX beamlines, it has been observed that some crystals are seen to ‘glow’ during X-ray data collection. Visible luminescence has been observed both from individual amino acids and proteins in solution (Carter *et al.*, 1965 ▶) but the signal has not been collected from cryo-cooled protein crystals. Using our online set-up, XEOL spectra were obtained from cryo-cooled bovine trypsin crystals at 100 K using an excitation energy of 13.3 keV. The complex spectra which are observed result from the interaction of X-rays at energies close to the absorption edge of elements in the protein crystal and surrounding solution. Multiple overlapping bands are a feature of the emission of optically excited states in a complex mixture of organic molecules and this signature is dependent on the excitation energy. In addition, diffusion of secondary electronic excitations and self-absorption of the optical emission by the sample can also contribute to the spectrum.

XEOL is distinct from ‘standard’ UV/VIS fluorescence and represents a complex multi-electron process that has been described as an ‘X-ray photon in and optical photon out event’ (Rogalev & Goulon, 2002 ▶). Studies using soft X-rays as the excitation source are well documented. However the detailed processes involved at higher energies (*i.e.* 13 keV) have not been previously described for proteins and require further study. XEOL using tunable soft X-rays has been successfully utilized to study proteins labelled with fluorescein-isothiocyanate (FITC), commonly used in biological immunofluorescence (Kim *et al.*, 2004 ▶). By exciting at the C, O and N *K*-edges, these studies revealed that carbon, but not oxygen or nitrogen, is coupled to the luminescence from the FITC chromophore. The simplicity of the XEOL experiment with the microspectrophotometer opens up the possibility of performing a range of new studies.

The device described in this paper is available at the ESRF and has been utilized over the last few years to collect UV/VIS and fluorescence data by over 30 groups, trained and supervised by various authors of this paper. A recent example of its use was reported by Badyal *et al.* (2008 ▶), where our instrument was used to probe iron oxidation states within the active site of a heme peroxidase. Comparison of solution and crystal spectra allowed confirmation that no structural effects resulted from cryo-protection and cryo-cooling. Online UV/VIS measurements aided in the interpretation of the MX data during significant radiation-induced haem reduction. Subtle differences in the X-ray-reduced and chemical-reduced spectra could be rationalized in the context of the structural results and further demonstrate the application of the combined techniques. Another recent example of the use of the instrument can be found by Macedo *et al.* (2009 ▶) in which a combination of UV/VIS microspectrophotometry and X-ray crystallography has been used to test the efficacy of selected scavengers in reducing the undesirable photoreduction of metalloproteins.

The online microspectrophotometer was initially conceived as a tool that, by measuring the production of optically absorbing species known to be radiolytic products, could provide a radiation damage metric that would guide diffraction data collection. Unfortunately, it is now clear that the rate of radical production is much faster than the crystallographically observable damage to the crystals. Notwithstanding this realisation, the device has become a very welcome addition to the armoury of techniques that can yield complementary evidence to elucidate three-dimensional snapshots of macromolecules at work. Its full potential has yet to be realised.

## Footnotes

1
               http://www.esrf.eu/UsersAndScience/Experiments/MX/How_to_use_our_beamlines/Run_Your_Experiment/Microspectrophotometer_User_Guide.
